# Supplementary material for: Decriminalizing suicide: the 2017 Mental Healthcare Act and suicide mortality in India, 2001–2020
Source: Glob Ment Health (Camb). 2025 Jun 30;12:e74. doi: 10.1017/gmh.2025.10031 (PMC12277205; doi:10.1017/gmh.2025.10031)

**Appendix**

**Table A1.** Fixed effects Poisson regression results predicting outlier-adjusted change in suicides (with a population offset) as a function of the decriminalization of suicide in 16 less developed Indian states, 2001-2020.

|  | **Less developed states** |
| --- | --- |
| **Covariates** | **IRR (95% CI)** |
| Decriminalization of suicide |  |
| Before 2018 | -- |
| 2018 and after | 1.856 (1.028 – 3.363)** |
| Infant mortality (per 1,000 live births) | 1.004 (0.997 – 1.011) |
| Gross state domestic product per capita (Indian rupees) | 1.000 (0.999 – 1.000) |
| Literacy rate (%) | 0.978 (0.946 – 1.011) |
| N (# of states) | 310 (16) |

*p<0.1; **p<0.05; ***p<0.001

N represents state-year combinations

State and year indicators included but not shown

**Table A2.** Fixed effects Poisson regression results predicting change in suicides among males and females (with a population offset) as a function of the decriminalization of suicide in 35 Indian states, 2001-2020.

|  | **Male suicides** | **Female suicides** |
| --- | --- | --- |
| **Covariates** | **IRR (95% CI)** | **IRR (95% CI)** |
| Decriminalization of suicide | 1.293 (0.582 - 2.874) | 0.645 (0.346 - 1.202) |
| Infant mortality (per 1,000 live births) | 0.999 (0.985 - 1.012) | 0.995 (0.985 - 1.004) |
| Gross state domestic product per capita (Indian rupees) | 1.000 (0.999 - 1.000) | 0.999 (0.999 - 1.000) |
| Literacy rate (%) | 0.992 (0.948 - 1.039) | 1.001 (0.965 - 1.039) |
| N (# of states) | 620 (31) | 620 (31) |

*p<0.1; **p<0.05; ***p<0.001

N represents state-year combinations

State and year indicators included but not shown

**Table A3.** Fixed effects Poisson regression results predicting change in suicides among males and females (with a population offset) as a function of the decriminalization of suicide in 16 less developed Indian states, 2001-2020.

|  | **Less developed states** | |
| --- | --- | --- |
|  | **Male suicides** | **Female suicides** |
| **Covariates** | **IRR (95% CI)** | **IRR (95% CI)** |
| Decriminalization of suicide | 2.701 (1.329 – 5.492)** | 0.845 (0.430 – 1.663) |
| Infant mortality (per 1,000 live births) | 1.006 (0.997 – 1.015) | 0.998 (0.991 – 1.006) |
| Gross state domestic product per capita (Indian rupees) | 1.000 (0.999 – 1.000) | 1.000 (0.999 – 1.000) |
| Literacy rate (%) | 0.973 (0.935 – 1.013) | 0.989 (0.963 – 1.016) |
| N (# of states) | 320 (16) | 320 (16) |

*p<0.1; **p<0.05; ***p<0.001

N represents state-year combinations

State and year indicators included but not shown

**Table A4.** Fixed effects Poisson regression results predicting change in accidental deaths and homicides among (with a population offset) as a function of the decriminalization of suicide in 16 less developed Indian states, 2001-2020.

|  | **Accidental deaths** | **Homicides** |
| --- | --- | --- |
| **Covariates** | **IRR (95% CI)** | **IRR (95% CI)** |
| Decriminalization of suicide | 0.067 (0.007 – 0.614)** | 0.024 (0.004 – 0.179)*** |
| Infant mortality (per 1,000 live births) | 0.997 (0.976 – 1.019) | 0.988 (0.972 – 1.004) |
| Gross state domestic product per capita (Indian rupees) | 1.000 (1.000 – 1.000)** | 1.000 (1.000 – 1.000)*** |
| Literacy rate (%) | 1.096 (1.018 – 1.180)** | 1.070 (1.020 – 1.122)** |
| N (# of states) | 304 (16) | 320 (16) |

*p<0.1; **p<0.05; ***p<0.001

N represents state-year combinations

State and year indicators included but not shown

**Figure A1.** Classification of States and Union Territories in India by level of development

**
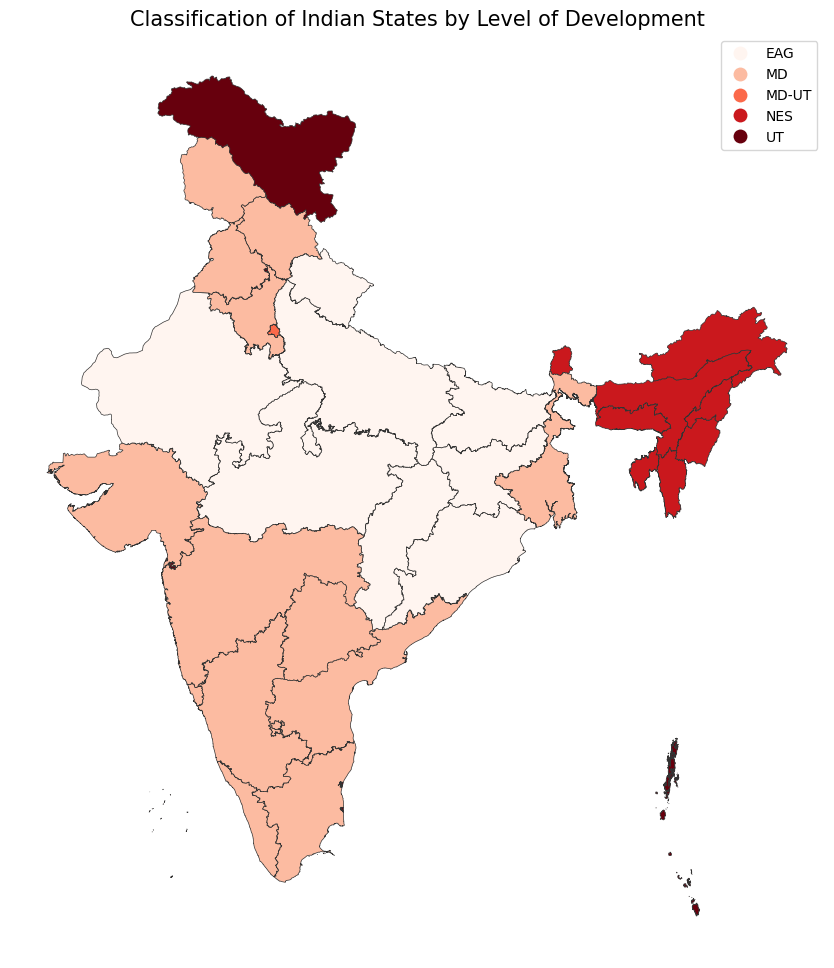
**

Note: EAG – Empowered Action Group States Group (Bihar, Chhattisgarh, Jharkhand, Madhya Pradesh, Odisha, Rajasthan, Uttar Pradesh and Uttarakhand); NES – Northeast States (Arunachal Pradesh, Assam, Manipur, Meghalaya, Mizoram, Nagaland, Sikkim, and Tripura); MD – More Developed States (Andhra Pradesh, Goa, Gujarat, Haryana, Himachal Pradesh, Karnataka, Kerala, Maharashtra, Punjab, Tamil Nadu, Telangana, West Bengal) ; UT – Union Territories (Dadra and Nagar Haveli and Daman & Diu, Jammu & Kashmir, Ladakh., Chandigarh., Puducherry, Lakshadweep., Andaman and Nicobar Islands); MD-UT – More Developed Union Territory (Delhi).

**Figure A2.** Suicides (per 100,000 population) among men and women in all 35 states and Union Territories in India (A) and by level of state development (B) from 2001 – 2020. The vertical line indicates the decriminalization of suicide in 2018.

1. All states


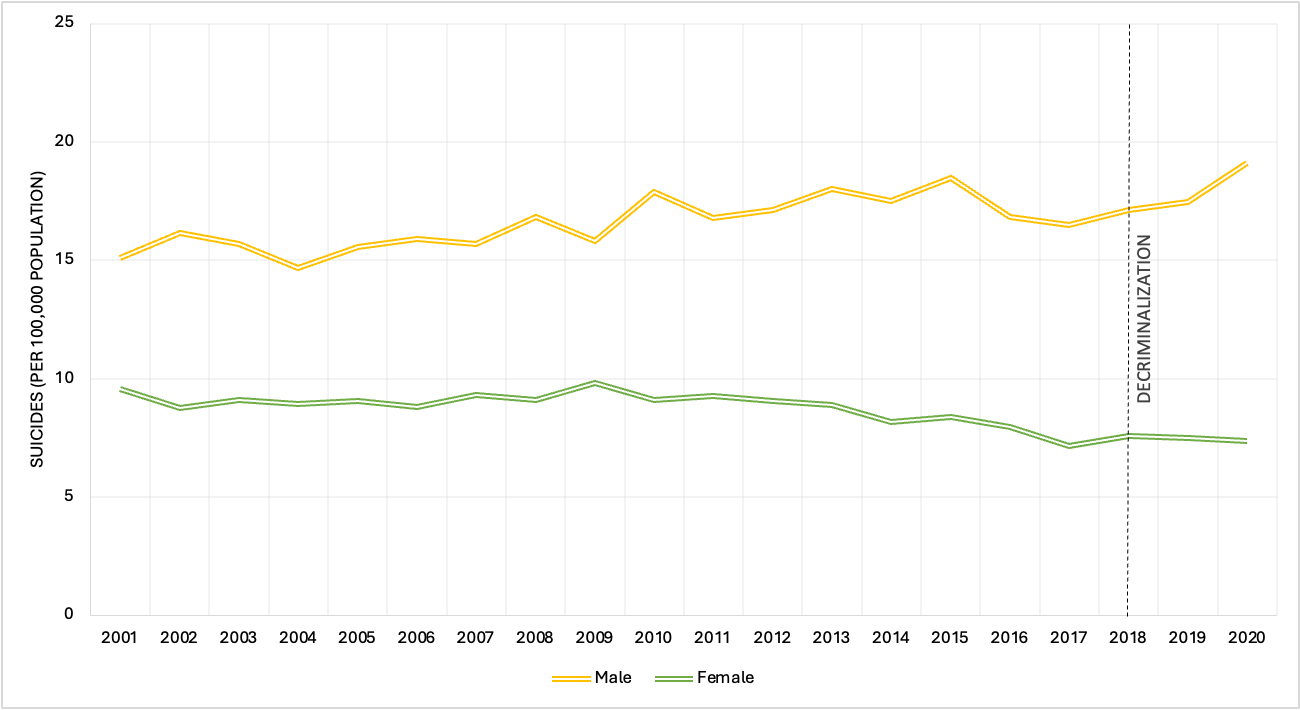


1. By level of state development


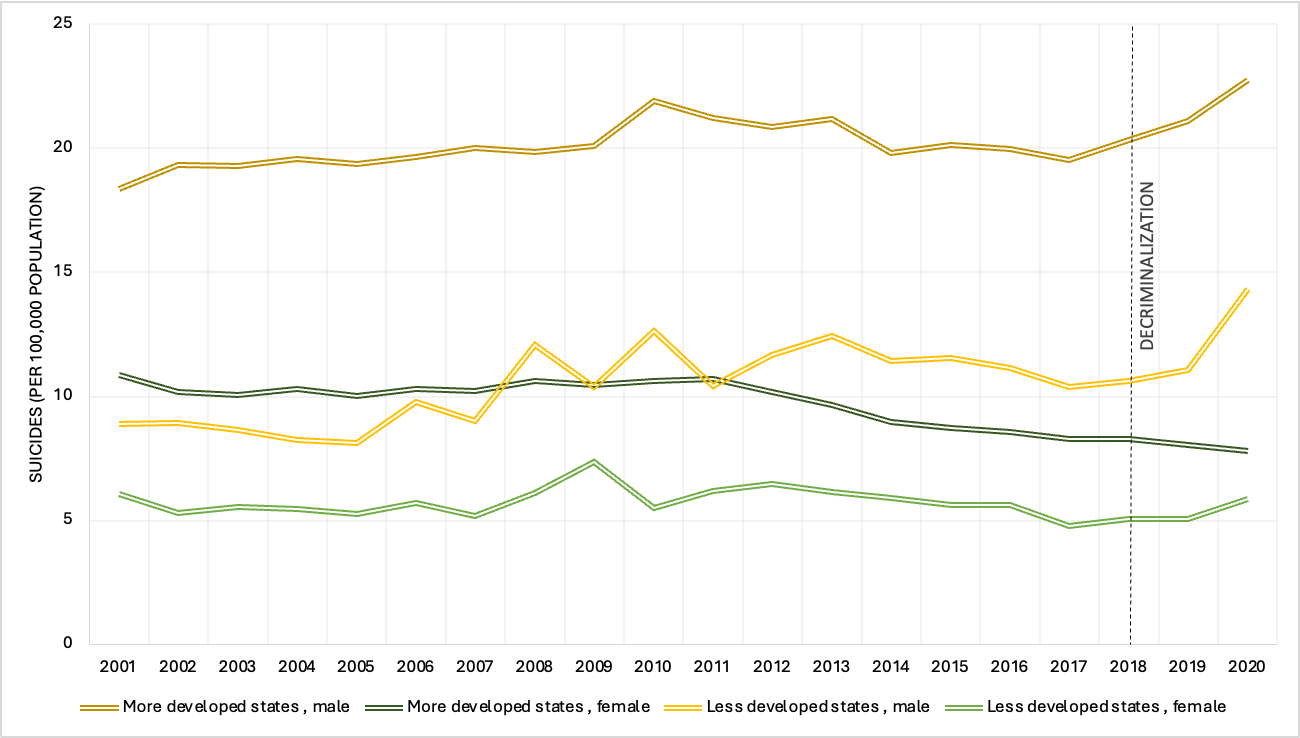

Supplement: Ganguli et al. supplementary material [file S2054425125100319sup001.docx]
